# Challenges in diagnosing and managing hyper-IgE syndrome in a resource-limited setting: a case report

**DOI:** 10.1097/MS9.0000000000002407

**Published:** 2024-07-25

**Authors:** Pratik Adhikari, Rabin Regmi, Pramodman Singh Yadav, Sujan Kafle

**Affiliations:** a B.P. Koirala Institute of Health Sciences, Dharan; b Patan Academy of Health Sciences, Lalitpur, Nepal

**Keywords:** elevated immunoglobulin E levels, hyper-IgE syndrome, recurrent infections, resource-limited setting

## Abstract

**Introduction and importance:**

Hyper-IgE syndrome (HIES), also known as Job syndrome, is a rare immunodeficiency disorder characterized by elevated immunoglobulin E levels and recurrent infections. Diagnosing and managing HIES in resource-limited settings is challenging due to the lack of advanced diagnostic tools. This report highlights the necessity of clinical evaluation and basic laboratory investigations for diagnosing HIES.

**Case presentation:**

A 3-year-old male presented with fever, cough, and widespread pustular lesions. He had a history of recurrent respiratory infections and otitis media. Physical examination revealed characteristic facial features, skin findings, and laboratory investigations showed elevated immunoglobulin E levels (>3000 IU/ml) and leukocytosis. A clinical diagnosis of HIES was made, and the patient responded well to antibiotics, antihistamines, and topical steroids.

**Clinical discussion:**

HIES is caused by genetic mutations affecting immune function, primarily involving STAT3 and DOCK8 genes. Diagnosis in resource-limited settings relies on clinical features and basic investigations. Challenges include the unavailability of genetic testing. Management includes antibiotics and symptomatic relief adapted to available resources.

**Conclusion:**

Diagnosing and managing HIES in resource-limited settings requires adaptation of clinical approaches to available resources. This case underscores the importance of clinical vigilance and basic diagnostic tools in diagnosing rare immunodeficiencies.

## Background

Highlights

- This case report discusses the diagnosis and management of hyper-IgE syndrome in a 3-year-old male with recurrent infections and elevated immunoglobulin E levels.
- The diagnosis was made clinically and supported by basic laboratory tests, as genetic testing was not available in this resource-limited setting.
- Treatment involved intravenous antibiotics, antihistamines, and topical steroids, resulting in significant clinical improvement.
- The report underscores the necessity of adapting management strategies to available resources and highlights the need for enhanced diagnostic facilities in under-resourced areas.

Hyper-IgE syndrome (HIES), also known as Job syndrome or Buckley syndrome, is a rare primary immunodeficiency disorder characterized by abnormally high levels of immunoglobulin E (IgE) antibodies in the blood. It is caused by genetic mutations affecting the immune system’s ability to fight off infections, leading to recurrent and severe bacterial and fungal infections^1^. The incidence of HIES in the world is estimated to be around 1 in 1 million people, and its incidence in Nepal is not well studied^2^. The classic clinical features of HIES are referred to as the “triad,” which includes recurrent staphylococcal skin abscesses, recurrent pneumonia, and elevated serum IgE levels, as seen in the reported case below. Other clinical features may include eczema, skeletal abnormalities, and dental abnormalities.

HIES is caused by multiple gene defects, some of which are still being studied, and the pathogenesis is not well known. Common genetic mutations associated with HIES include STAT3 and DOCK8 deficiencies. STAT3 mutations are typically linked to the autosomal dominant (AD) form of HIES, while DOCK8 mutations are associated with the autosomal recessive form^3–5^. These genetic defects result in impaired immune responses, particularly in the Th17 cell pathway, which plays a crucial role in mucosal immunity and inflammation^6^. Understanding the molecular and genetic basis of HIES is essential for accurate diagnosis and management.

A thorough clinical evaluation, combined with appropriate laboratory investigations and supportive care, can aid in the diagnosis and management of this challenging condition in resource-limited settings. Recent studies emphasize the importance of early genetic testing and molecular diagnostics to confirm HIES, as well as the potential benefits of targeted therapies and immunomodulatory treatments^7^.

## Case presentation

A 3-year-old male child from non-consanguineous parents presented to the Pediatrics Outpatient Department with fever, cough, and pustular lesions involving the whole body. The child had been admitted multiple times (3–4 times a year) for lower respiratory tract infections and recurring episodes of otitis media since infancy.

The child had an uneventful birth history and was immunized according to the standard immunization schedule. There were no similar episodes in the family and no history of asthma, tuberculosis, diabetes mellitus, hypertension, or atopy.

The child presented with a sudden onset of rashes on his face, which progressed downwards, involving the whole body. These rashes were red, weepy, crusty, itchy, flaky patches, shaped like oval or circular areas with multiple healed scars, including those on the genitalia. Additionally, there were pustules associated with swelling of the face and hands. Initial treatment with a local ointment was ineffective, and the fever persisted, temporarily relieved by paracetamol (15 mg/kg) but recurring after the medication’s effects weaned off.

On examination, the child exhibited swelling on the face and forefinger that was solid, non-tender, and not warm to touch, with minor desquamation and peeling skin over the soles and palms. The child’s vital signs included a temperature of 38.5°C, heart rate of 120 beats per min, respiratory rate of 28 breaths per min, blood pressure of 90/60 mm Hg, and oxygen saturation of 98% on room air. The child had a distinct facial appearance characterized by a broad nasal bridge, depressive asymmetric face, mild prognathism, and coarse features. The child’s weight was 7 kg and height was 76 cm, both below the third percentile, indicating Failure to Thrive. Additionally, the child had a pale complexion, short stature (147 cm), and no signs of puberty (Tanner Stage I). Oral thrush was absent, and teeth examination was normal. There was no lymphadenopathy or hepatosplenomegaly.

**Figure 1:**
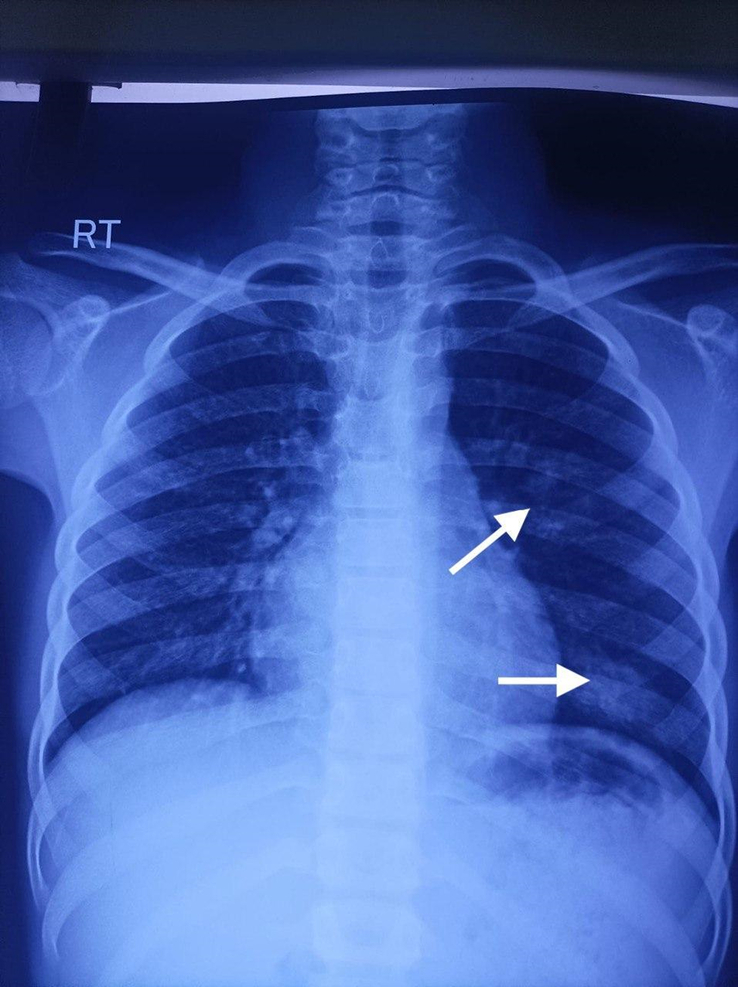
Chest X-ray showing infiltrations in the middle and lower zones of the left lung as shown by arrows.

**Figure 2:**
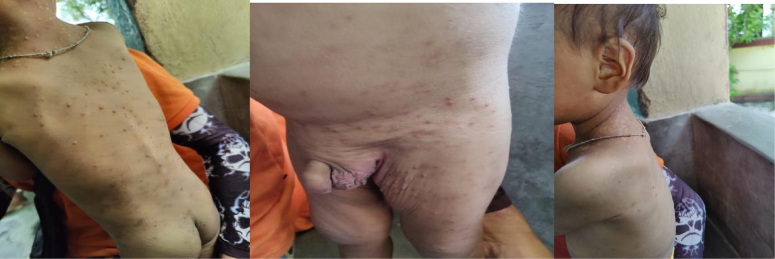
It demonstrates the pustular lesions with multiple healed scars involving the whole body including genitalia.

Laboratory investigations revealed a total leukocyte count of 16 750/cu mm, hemoglobin of 9.3 g/dl, and a peripheral blood smear showing a microcytic, hypochromic blood picture with leukocytosis and mild thrombocytosis (Table 1). Serum protein electrophoresis indicated an increased IgE level of >3000 IU/ml and the Mantoux test was negative (Table 2). A chest X-ray revealed infiltrations in the middle and lower zones of the left lung as shown by arrows (Fig. 1). Additionally, clinical photographs documented pustular lesions with multiple healed scars involving the whole body, including the genitalia (Fig. 2). The iron profile was also assessed and detailed in Table 2.

**Table 1:** General laboratory investigations

Test	Result	Reference range
Total leukocyte count	16 750/cu mm (elevated)	4000–11 000/cu mm
Neutrophil	61%	40–75%
Lymphocyte	33%	20–45%
Monocyte	4%	2–10%
Eosinophils	2%	1–6%
Hemoglobin	9.3 g/dl (low)	12–18 g/Dl
Platelet count	475 000/cu mm (elevated)	150 000–400 000/cu mm
C-reactive protein	Positive	Positive
Iron	26.0 mcg/dl (low)	65.0–175.0 mcg/dl
TIBC	251.0 mcg/dl	250–450 mcg/dl
Ferritin	>1650.0 ng/dl (high)	7–140 ng/dl

TIBC, total iron binding capacity.

**Table 2:** Immunological investigations

Test	Result	Reference range
IgE	>3000 IU/ml (elevated)	0.4–351.6 IU/ml
Mantoux test	5 mm after 72 h	0–15 mm
Serum protein electrophoresis	Elevated IgE	Normal

IgE, immunoglobulin E.

Based on the clinical presentation, recurrent infections, elevated IgE levels, and characteristic physical findings, a diagnosis of HIES was made. The chest X-ray findings supported this diagnosis. Due to resource limitations, genetic testing was not performed, and the diagnosis was made clinically and supported by laboratory findings.

In the hospital, the patient was treated for 2 weeks. Management included intravenous ceftriaxone (50 mg/kg/d in divided doses) and clindamycin (20 mg/kg/d in divided doses), oral antihistamines (diphenhydramine 1 mg/kg/dose every 6 h), and topical steroids (hydrocortisone 1% cream applied twice daily). The child’s fever subsided, and the rashes improved significantly.

This case highlights the challenges and considerations involved in diagnosing and managing HIES in resource-limited settings. The diagnosis primarily relied on the characteristic clinical features and basic investigations like blood tests and chest X-ray provided additional support. Management strategies were adapted to available resources, and educating the parents about HIES, along with encouraging them to seek care at a more specialized facility if possible in the future, was crucial.

I am writing in accordance with the SCARE checklist. In accordance with the SCARE 2023 guideline (Sohrabi *et al.*, 2023), the methodology for reporting surgical case details was strictly adhered to in this study^8^.

## Discussion

In 1966, the syndrome was first described as “Job’s syndrome” by Davis *et al.* ^9^ in two girls suffering from recurrent “cold” staphylococcal abscesses, pneumonia, and neonatal-onset eczematoil rash, referring to the Biblical Job, who was “smote with sore boils” The disease was later reported as HIES by Buckley and colleagues in 1972 because they found that these symptoms were associated with exceptionally high serum concentrations of IgE^10^.

HIES is a rare immunodeficiency syndrome of which the exact pathogenesis is still unknown. There is no specific clinical and laboratory test for confirming. Several symptoms such as elevated IgE levels and eosinophilia might also be found in other immunodeficiency syndromes^2^. Therefore, we must synthetically analyze the medical history, appearance and skin characteristics, visceral abnormalities, atypical presentations and necessary laboratory study findings including cytokines and immunoglobulins levels. Our patient’s atypical presentation was not working out until all the above mentioned aspects were examined. And all the symptoms indicated that he is a patient with HIES.

The diagnosis of HIES is difficult to be confirm in that both immunologic and somatic features need to be identified prior to genetic testing. There are two forms of HIES^11^. They have different pathogenesis, processes, and outcomes, and the only common ground is the IgE elevation, with values reaching 2000 IU (normal IU)^9,12^. Type 1 HIES, a dominant form caused by hypomorphic mutations in STAT3, is a disease of multi-organ dysfunction. Besides eczema and recurrent staphylococcal infections in skin and lung, these patients suffer from abnormalities in vessels, connective tissue, and skeleton^4^. STAT3 (signal transducer and activator of transcription 3) is located on human chromosome 17q21, which was reported to contain a disease locus for familial AD- HIES^12^. It is a transcription factor, which binds to the STAT3-responsive elements in the promoters of various genes and plays a critical role in responses to many cytokines, in which, IL-17 produced by 17 cell is protective in the host defense against extracellular bacteria^6^, and IL-22 stimulates cells in the skin and respiratory systems to produce defensins through STAT3 activation^3^. Therefore, the HIES etiology might be directly and indirectly linked to STAT3. In other words, a human deficiency in STAT3 is a major cause of sporadic and familial HIES. The type 2 HIES is autosomal recessive syndrome^13^. The patients with type 2 HIES did not show any skeletal and dental abnormalities, and had no pulmonary cysts, but most of them suffered from viral infections such as chronic refractory Molluscum Contagiosum and Herpes Simplex virus infections, which were not identified in type 1 HIES.

In one study on a 14-year-old boy in Iran, asthma and anaphylaxis were reported as two atypical presentations in an AD‐HIES patient suffering from dental abnormalities, eczema, and recurrent sinusitis^14^.

Our patient had multiple features suggestive of HIES. His typical facies and high serum IgE levels together with eosinophilia, and skin infections were highly suggestive of HIES. The absence of skeletal and dental pathology indicates autosomal recessive pattern of inheritance; however, genetic analysis is needed for confirmation^15^. Due to resource limitations, genetic testing was not performed, highlighting a significant challenge in diagnosing and managing HIES in developing countries.

In resource-limited settings, the lack of advanced diagnostic tools like genetic testing poses a significant barrier to diagnosing primary immunodeficiencies such as HIES. This issue is compounded by the broader challenges in healthcare systems, including limited access to specialized care and insufficient awareness about rare immunodeficiency disorders among healthcare providers. Studies have highlighted these barriers, emphasizing the need for improved diagnostic facilities and training in resource-limited regions^16^.

The difficulties in diagnosing HIES are indicative of broader issues faced in diagnosing less severe and less difficult immune-mediated diseases in developing countries. These challenges underscore the importance of clinical evaluation and basic laboratory investigations in making a diagnosis. Moreover, there is a need for ongoing research and development of cost-effective diagnostic tools to improve the identification and management of such conditions^17,18^.

In summary, our case highlights the necessity of adapting management strategies to available resources and the importance of clinical vigilance in diagnosing HIES in resource-limited settings. Enhanced diagnostic facilities and a greater emphasis on education and awareness about primary immunodeficiencies are crucial for improving patient outcomes in these regions.

## Conclusion

Diagnosing and managing HIES in resource-limited settings is challenging due to limited access to advanced genetic testing and comprehensive laboratory investigations. Recognizing characteristic clinical features and using basic diagnostic tools, such as blood tests and chest X-rays, are crucial. Effective management involves antibiotics, symptomatic relief, and patient education. This case emphasizes the need for adapting healthcare approaches to available resources and improving diagnostic facilities in under-resourced regions.

## Ethical approval

Not applicable.

## Consent

Informed consent was taken from the patient to publish this case report.

## Source of funding

Not applicable.

## Author contribution

P.A. and R.R. provided with data and materials from the archive and their notes. P.S.Y., S.F. and R.R. wrote the manuscript, collected the images and put them in perspective according to the timeline of the case. P.A. reviewed the manuscript and did final editing.

## Conflicts of interest disclosure

The authors declare no conflicts of interest.

## Research registration unique identifying number (UIN)

This is a cross-sectional involving a human subject, so registration of research study was done.

1. Registry used: Researchregistry.com.

2. Unique Identifying number or registration ID: researchregistry10377.

## Guarantor

Pratik Adhikari is the guarantor of the study.

## Data availability statement

The datasets supporting the conclusions of this article are included within the article.

## Provenance and peer review

Not commissioned or externally peer-reviewed.
